# Thrombus formation after the Norwood procedure: Incidence, risk factors, and its impact on late outcomes^☆^

**DOI:** 10.1016/j.ijcchd.2025.100575

**Published:** 2025-02-15

**Authors:** Alessandra Poppe, Muneaki Matsubara, Jonas Palm, Thibault Schaeffer, Takuya Osawa, Carolin Niedermaier, Paul Philipp Heinisch, Nicole Piber, Bettina Ruf, Alfred Hager, Peter Ewert, Jürgen Hörer, Masamichi Ono

**Affiliations:** a Department of Congenital and Pediatric Heart Surgery, German Heart Center Munich, Technische Universität München, Munich, Germany; b Division of Congenital and Pediatric Heart Surgery, University Hospital of Munich, Ludwig- Maximilians-Universität München, Munich, Germany; c Europäisches Kinderherzzentrum München, Munich, Germany; d Department of Congenital Heart Disease and Pediatric Cardiology, German Heart Center Munich, Technische Universität München, Munich, Germany; e Department of Cardiovascular Surgery, German Heart Center Munich, Technische Universität München, Munich, Germany

**Keywords:** Thrombus, Hypoplastic left heart syndrome, Norwood procedure, Restrictive atrial septal defect

## Abstract

**Objective:**

Thrombus formation is a feared complication after congenital heart surgery. We aimed to clarify the clinical characteristics associated with thrombus formation after the Norwood procedure.

**Methods:**

All neonates who underwent the Norwood procedure between 2001 and 2022 were reviewed. The incidence and location of thrombus were evaluated. Risk factors for thrombus formation and its impact on survival were analyzed.

**Results:**

Among 360 patients who were included, thrombus formation was detected in 42 patients (11.7 %) during the postoperative in-hospital period, with a median of 12 (range: 5–30) postoperative days. The most common site of thrombus was the superior vena cava in 9 (2.5 %) patients, followed by the right atrium in 8 (2.2 %). Patients who received a right ventricle to pulmonary artery conduit had a higher incidence of thrombus than those who received a modified Blalock-Taussig-Thomas shunt (16.4 vs. 7.7 %, p = 0.011). Patients with thrombus formation had a longer stay in the intensive care unit (ICU), than those without (median 21 vs. 13 days, p = 0.018). Survival after ICU discharge was lower in patients with thrombus than those without (57, 54, and 54 % vs 73, 71, and 70 % at 2, 4, and 6 years, respectively; p = 0.032). Restrictive atrial septal defect was identified as an independent risk for thrombus (odds ratio: 2.61; p = 0.005).

**Conclusions:**

Thrombus formation was observed in 12 % of the patients during the hospital stay after the Norwood procedure and was associated with prolonged recovery and high mortality. A restrictive atrial septal defect was identified as a risk factor for thrombus formation.

## Highlights

• Thrombus formed in 11.7 % of patients after Norwood procedure, mainly in the superior vena cava.
• Restrictive atrial septal defect emerged as an independent risk factor for thrombus formation.
• Six-year survival was lower in patients with thrombus (54 %) compared to those without (70 %).

## Abbreviations and Acronyms

ASD: atrial septal defect
APTT: activated partial thromboplastin time
BCPS: bidirectional cavopulmonary shunt
CI: confidence interval
ECMO: extra-corporeal membrane oxygenation
HLHS: hypoplastic left heart syndrome
ICU: intensive care unit
IQR: interquartile ranges
MBTTS: modified Blalock-Taussig Thomas shunt
OR: odds ratio
PT-INR: prothrombin time international normalized ratio
RVPAC: right ventricle-to-pulmonary artery conduit
SVC: superior vena cava
TCPC: total cavopulmonary connection

## 1 Introduction

Hypoplastic Left Heart Syndrome (HLHS) and its variants represent one of the most challenging and high-risk congenital heart diseases to be surgically managed. Despite significant advancements in surgical techniques and perioperative management, the neonatal Norwood procedure, typically the first stage of surgical palliation, still faces plateauing early survival rates over the last decades [1]. This has led to extensive research into risk factors for mortality following the Norwood procedure, with thrombosis emerging as a particularly severe complication associated with increased morbidity and mortality [2]. Children undergoing cardiac surgery, especially those with single ventricle physiology like HLHS, are at heightened risk for thrombus formation due to multiple factors. Disruption of blood flow due to surgery, changes in platelet function, inflammation, and hypercoagulable states can all contribute to thrombus formation [3]. The immature coagulation system of neonates further increases this risk due to a decrease in antithrombotic ability and unpredictable reactions to anticoagulant therapy.

Our study aims to evaluate the incidence of post-Norwood thrombus formation, its impact on mortality and progression through subsequent palliative stages, and to identify the predisposing risk factors for thrombus formation.

## 2 Methods

### 2.1 Data availability

The corresponding author will share the data underlying this article upon reasonable request.

### 2.2 Ethical statement

The study was approved by the Institutional Review Board of the Technical University of Munich (approval number 2024-334-S-CB on July 08, 2024). Due to the retrospective nature of the study, the requirement for individual patient consent was waived.

### 2.3 Patients and data collection

This single-center retrospective study reviewed all neonates with HLHS and its variants who underwent the Norwood procedure at the German Heart Center Munich between 2001 and 2022. Patients who received the Norwood procedure beyond the neonatal period (after 28 days of life) and those who underwent bilateral pulmonary artery banding were excluded. Patient baseline and follow-up information was acquired from our single ventricle database, which encompasses outcomes of the subsequent palliative stages, including the bidirectional cavopulmonary shunt (BCPS) (Stage II) and the total cavopulmonary connection (TCPC) for Fontan completion (Stage III). The follow-up period for each patient was defined as the interval between the initial Norwood procedure and the most recent clinical examination. For deceased patients, follow-up was censored at the time of death.

### 2.4 Surgical techniques and perioperative management

The surgical techniques for the Norwood procedure were previously described in detail [4]. The choice between a modified Blalock-Taussig-Thomas shunt (MBTTS) or right ventricle to pulmonary artery conduit (RVPAC) was made based on surgeons’ preference. In patients who received a 3.5 mm heparin-coated Gore-Tex tube was used most frequently, a 3.0 mm shunt was used in patients weighing less than 2.5 kg, and a 4.0 mm shunt was used in selected patients. The proximal and distal anastomoses were placed in the brachiocephalic artery and in the right PA, respectively. In patients who received a non-ringed Gore-Tex conduit (5 mm) was used before 2005. Since 2006, a 5-mm or 6-mm ring-reinforced Gore-Tex conduit has been used. The proximal anastomosis was performed by conventional method until 2011, after which the dunk technique was adopted in 2012. For the distal anastomosis, the RVPAC was positioned to the right of the neo-ascending aorta (>95 % of patients).

Postoperative thrombosis prophylaxis was standardized across all patients. Unfractionated heparin was administered intravenously at a dose of 5000 IU/m^2^/day. The target partial thromboplastin time was set at 60 s. This regimen was maintained until all central vascular lines were removed, with a 4.5 Fr. catheter typically being utilized. Thereafter, patients with a shunt diameter of 4 mm or less received acetylsalicylic acid (3–5 mg/kg/day), and patients with a shunt diameter of more than 4 mm had no further anticoagulation.

### 2.5 Detection of thrombus

Postoperative thrombus was primarily detected using transthoracic echocardiography, with angiography employed in select cases. Echocardiographic examinations were performed using a Vingmed ultrasonographic system (GE Vingmed Ultrasound AS; Strandpromenaden, Horten, Norway) equipped with 5.0-, 3.5-, or 2.5-MHz phased-array transducers. Cardiac catheterization and angiography were conducted for patients exhibiting problematic hemodynamics. When performed, these procedures included pulmonary artery angiography, aortography, and systemic venous angiography to evaluate for the presence of a thrombus. In this study, a thrombus was defined as any localized echogenic mass detected within the heart or any extra-cardiac location close to the heart. Thrombus found outside the blood vessels, including those removed from the chest cavity, mouth, trachea, or thoracic drainage tube, were excluded. Thrombus associated with central vascular lines and those that occurred within the extracorporeal membrane oxygenation (ECMO) circuit were also excluded from the analysis of this study.

### 2.6 Echocardiography

Echocardiographic assessments were described in our previous report [5]. An experienced echocardiographer calculated the ejection fraction (EF), and the atrioventricular valve regurgitation was graded by analyzing the width and length of the regurgitant jet (none, mild, moderate, severe). Ventricular dysfunction was defined as an EF below 50 %. Significant atrioventricular valve regurgitation was diagnosed when graded as moderate or more severe. A restrictive atrial septal defect (ASD) was defined according to the findings of the pressure gradient between the left atrium and the right atrium of more than 8 mmHg.

### 2.7 Statistical analysis

Categorical variables were presented as numbers and percentages, while continuous variables were expressed as medians with interquartile ranges (IQR) or ranges. Chi-square tests were employed for categorical data analysis. For continuous variables, independent sample t-tests were utilized for normally distributed data, while Mann-Whitney U-tests were applied to non-normally distributed variables. Survival was calculated using the Kaplan-Meier method, with differences between groups determined via log-rank test. Risk factors for thrombus formation were initially assessed using logistic regression models. Statistical significance was set at p klein. All statistical analyses were performed using SPSS version 28.0 for Windows (IBM, Ehningen, Germany) and R-statistical software (R Foundation for Statistical Computing, Vienna, Austria).

## 3 Results

### 3.1 Patient characteristics

A total of 360 neonates underwent the Norwood procedure during the study period. Thrombus formation was observed in 42 patients (11.7 %) during the hospital stay following the Norwood procedure, at the median time of 12 days (range: 5–30 days). Patient characteristics with or without thrombus formation are shown in Table 1. HLHS was presented in 86 % of the patients, and patients with HLHS had a higher incidence of thrombus formation than those with other types of functional single ventricle that underwent a modified Norwood procedure (92.6 % vs 86.6 %, p = 0.035). A higher rate of thrombus formation was observed in patients with restrictive ASD compared to those with non-restrictive ASD (57.1 % vs 31.1 %, p < 0.001).

**Table 1:** Baseline cohort characteristics.

Variables: N (%) or median (IQR)	Total	Thrombus(+)	Thrombus(−)	p-value
Number of patients	360	42	318	
Male sex	248 (68.9)	28 (66.7)	220 (69.2)	0.741
Gestational age (wk)	39 (38–40)	39 (37–40)	39 (38–40)	0.654
Birth weight (kg)	3.2 (2.9–3.5)	3.2 (2.7–3.6)	3.2 (2.9–3.5)	0.231
Genetic anomaly	17 (5.1)	1 (2.4)	16 (5.5)	0.410
Extracardiac anomaly	45 (13.5)	5 (12.2)	40 (13.7)	0.798
**Primary Diagnosis**				
HLHS	291 (80.8)	39 (92.9)	252 (79.2)	0.035
Tricuspid atresia	17 (4.7)	2 (4.8)	15 (4.7)	0.990
DILV	29 (8.1)	1 (2.4)	28 (8.8)	0.151
UAVSD	15 (4.2)	0 (0.0)	15 (4.7)	0.150
**Associated anomaly**				
TGA	45 (12.5)	2 (4.8)	43 (13.5)	0.107
DORV	20 (6.0)	2 (4.9)	18 (6.1)	0.753
CoA	90 (26.9)	6 (14.6)	84 (28.6)	0.059
Dextrocardia	4 (1.1)	1 (2.4)	3 (0.9)	0.404
Heterotaxy	1 (0.3)	0 (0.0)	1 (0.3)	0.716
Dominant right ventricle	311 (86.4)	40 (95.2)	271 (85.2)	0.075
**Echocardiographic data**				
Ventricular dysfunction	42 (11.7)	4 (9.5)	38 (12.0)	0.636
Significant AVVR	21 (6.1)	4 (9.8)	17 (5.6)	0.301
Restrictive ASD	122 (34.2)	24 (57.1)	98 (31.1)	<0.001

HLHS: hypoplastic left heart syndrome, DILV: double inlet left ventricle; UAVSD: unbalanced atrioventricular septal defect; TGA: transposition of great arteries; DORV: double outlet right ventricle; CoA: coarctation of the aorta; AVVR: atrioventricular valve regurgitation; ASD: atrial septal defect.

### 3.2 Perioperative data

Table 2 shows perioperative variables and hospital data in patients with and without thrombus. A higher incidence of thrombus formation was observed in patients who received an RVPAC than those who received a MBTTS (64.3 vs 43.4 %, p = 0.011). The longer median length of stays was recorded for patients with thrombus formation in the ICU (21 vs. 13 days; p = 0.018) and hospital (35 vs. 23 days; p = 0.012) compared to those without thrombus formation. ECMO support was more frequently needed in patients with thrombus than those without (14 vs. 4 %, p = 0.003). Thrombus was detected in 18 patients (5 % of all patients) while on ECMO support. A higher proportion of patients with thrombus required ECMO than those without thrombus (14.3 vs. 3.8 %, p = 0.003). Hospital mortality was higher in patients with thrombus than those without (21 vs.1 %, p = 0.063).

**Table 2:** Perioperative variables and follow-up outcomes in patients with and without thrombus.

Variables: N (%) or median (IQR)	Total	Thrombus(+)	Thrombus(−)	p-value
Number of patients	360	42	318	
Age at Norwood (days)	8 (7–12)	8 (7–11)	8 (7–12)	0.449
Weight at Norwood (kg)	3.2 (2.9–3.5)	3.1 (2.7–3.6)	3.2 (2.9–3.5)	0.382
**Coagulation profile (Pre-Norwood procedure)**				
APTT (s)	43 (37–50)	42 (39–54)	43 (37–49)	0.336
PT-INR	1.2 (1.1–1.3)	1.1 (1.1–1.3)	1.2 (1.1–1.3)	0.559
Antithrombin (%)	63 (54–72)	62 (54–71)	63 (54–72)	0.468
Fibrinogen (mg/dL)	231 (200–286)	213 (178–240)	235 (201–288)	0.095
**Operative data**				
CPB time (min)	138 (109–165)	140 (118–183)	138 (107–163)	0.185
AXC time (min)	49 (41–59)	50 (41–59)	48 (40–59)	0.553
Lowest Temp (C)	19 (18–22)	19 (18–22)	19 (18–22)	0.670
**Shunt type**				
MBTTS	195 (47.2)	15 (35.7)	180 (56.6)	0.011
RVPAC	165 (45.8)	27 (64.3)	138 (43.4)	
**Postoperative data**				
ICU stay (days)	13 (8–21)	21 (14–41)	13 (8–20)	0.018
Hospital stay (days)	24 (15–37)	35 (23–83)	23 (15–36)	0.012
ECMO implantation	18 (5.0)	6 (14.3)	12 (3.8)	0.003
Hospital death during stage I	45 (12.2)	9 (21.4)	36 (11.3)	0.063
Reached Stage II	251 (69.7)	25 (59.5)	226 (71.0)	0.028
Fontan completion	191 (53.1)	17 (40.5)	174 (54.7)	0.032

APTT: activated partial thromboplastin time; PT-INR: prothrombin time international normalized ratio, CPB: cardiopulmonary bypass; AXC: aortic cross clamp; Temp: temperature; MBTTS: modified Blalock-Taussig Thomas shunt; RVPAC: right ventricle-to-pulmonary artery conduit; ICU: intensive care unit; ECMO: extra-corporeal membrane oxygenation.

### 3.3 Location of and treatment for thrombus

**Fig. 1:**
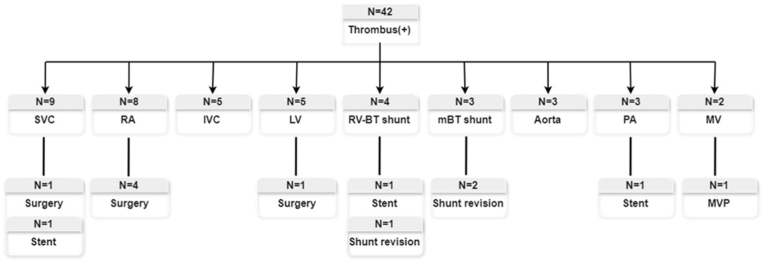
Location and thrombus therapy.

The locations of thrombosis are shown in Table 3. Intra-thoracic venous or atrial thrombus was found in 25 cases (6.9 %) and intra-thoracic arterial or ventricle thrombus in 17 cases (4.7 %), including shunt thrombus in 7 cases (1.9 %). The median time from Norwood procedure to detection of thrombus was 11 (range: 5–23) days for intra-thoracic venous thrombus, 15 (range: 4–46) days for intra-thoracic arterial thrombus, with 74 % of thrombus diagnosed within the first month following the Norwood procedure. The most frequent location was the superior vena cava (SVC) in 9 patients, followed by the right atrium in 8, the inferior vena cava in 5, and the left ventricle in 5. Thrombolytic therapy with tissue plasminogen activator, high-dose heparin, or Coumadin was used for all patients with thrombus. Detailed information regarding thrombosis sites and treatments is illustrated in Fig. 1. Surgical or interventional procedures were required in several cases: shunt revision was performed in 3 patients, stent placement in 3 patients, and surgical removal of thrombus in 7 patients. Additionally, one patient underwent concurrent mitral valvuloplasty during thrombus removal.

**Table 3:** Characteristics of thrombus in patients following the Norwood procedure (N = 360).

Variables: N (%) or median (IQR)	Number of patients	Time to onset of thrombus
		(from Norwood procedure, days)
**Intra-thoracic venous thrombus**	25 (6.9)	11 (5–23)
SVC	9 (2.5)	
RA	8 (2.2)	
IVC	5 (1.4)	
PA	3 (0.8)	
**Intra-thoracic arterial thrombus**	17 (4.7)	15 (4–46)
LV	5 (1.4)	
Aorta	3 (0.8)	
Mitral valve	2 (0.6)	
Shunt	7 (1.9)	
Total	42 (11.7)	12 (5–30)

SVC: superior vena cava, RA: right atrium, IVC: inferior vena cava, LV: left ventricle, PA: pulmonary artery.

### 3.4 Postoperative coagulation data

Activated partial thromboplastin time (APTT), prothrombin time international normalized ratio (PT-INR), antithrombin, and fibrinogen levels were analyzed preoperatively and on the postoperative days (POD) 1, 3, 7, and 14 (Supplementary Table S1). APTT levels on POD 7 and 14 were higher in patients with thrombus than those without (p = 0.007 and p = 0.027, respectively). Fibrinogen levels on POD 3 and 7 were lower in patients with thrombus than those without (p = 0.010 and p < 0.001, respectively).

### 3.5 Follow-up data

**Fig. 2:**
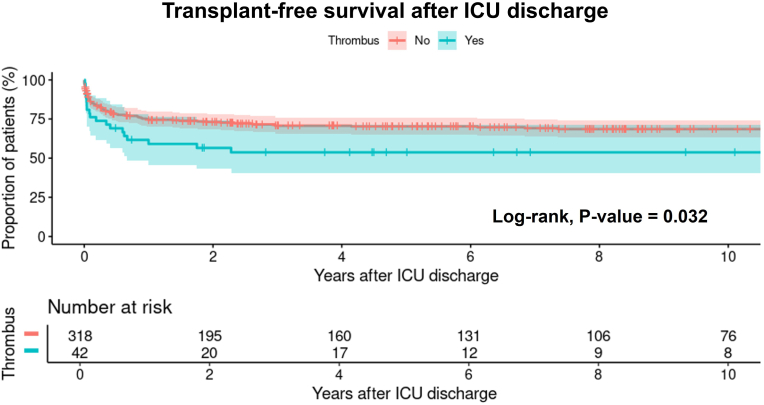
Transplant-free survival after ICU discharge stratified by thrombus formation.

The median follow-up period after the Norwood procedure was 3.8 years (interquartile range (IQR): 0.3–9.2 years), with a maximum follow-up of 22.7 years. The rate of reaching stage II (60 vs 71 %, p = 0.028) and of reaching Fontan (41 vs. 56 %, p = 0.032) was lower in patients with thrombus than those without. No patients received heart transplants during the follow-up, meaning the survival corresponds to the transplant-free survival. Transplant-free survival at 2, 4, and 6 years after ICU discharge were 56.5 % (95 % confidence interval (CI): 43.2–73.9 %), 53.7 % (95 % CI: 40.3–71.5 %), and 53.7 % (95 % CI: 40.3–71.5 %) respectively in patients with thrombus, compared to 73.1 % (95 % CI: 68.3–78.3 %), 70.7 % (95 % CI: 65.7–76.1 %), and 70.3 % (95 % CI: 65.2–75.7 %) in patients without thrombus (p = 0.032, Fig. 2).

### 3.6 Risk factors for thrombus formation

The results of the logistic regression model are presented in Table 4. In the univariate analysis, HLHS (odds ratio (OR): 3.97; p = 0.046), restrictive ASD (OR: 2.95; p = 0.009), and the RVPAC (OR: 2.35, P = 0.012) were identified as risk factors. Subsequently, a multivariate analysis revealed that restrictive ASD was identified as an independent risk factor for thrombus formation (OR: 2.61; p = 0.005).

**Table 4:** Risk factor analysis for thrombus formation after Norwood procedure.

Variables	Univariable			Multivariable		
	OR	(95 % CI)	P Value	OR	(95 % CI)	P Value
**Anatomical variables**						
HLHS	3.97	1.02–11.36	0.046			
**Echocardiographic data**						
Ventricular dysfunction	0.77	0.26-2.28	0.637			
Restrictive ASD	2.95	1.53-5.69	0.001	2.61	1.33-5.12	0.005
**Operative data**						
Weight at Norwood	0.74	0.38-1.45	0.381			
CPB time	1.00	0.99-1.01	0.187			
RV-PA shunt	2.35	1.20-4.58	0.012			

HLHS: hypoplastic left heart syndrome; ASD: atrial septal defect; CPB: cardiopulmonary bypass.

## 4 Discussion

In this study, the incidence of thrombus formation after the neonatal Norwood procedure was 12 %, with intrathoracic venous thrombus being more common than intrathoracic arterial thrombus. Thrombus formation was associated with prolonged hospital recovery and decreased survival after hospital discharge compared to patients without thrombus. Particularly, restrictive ASD was identified as a significant risk factor for thrombus formation.

### 4.1 Incidence of thrombus

The incidence of thrombus formation after the initial single ventricle palliation was reported to be 6–33 % [6]. However, few studies focused on the incidence of thrombus formation after the neonatal Norwood procedure. White et al. reported that the total incidence of thrombus formation after the Norwood procedure was 10.4 % (6.4 % during hospitalization and 4.0 % during inter-stage) [7]. On the other hand, Agarwal et al. reported in a systematic review of 15 cohort studies an incidence of thrombus ranging from 0 % to 40 % in patients who underwent the Norwood procedure [6]. Our study revealed a thrombus formation incidence of 11.7 %. The timing of thrombus formation after the initial single ventricle palliation was reported to be 0–30 days, but details are scarce [8]. The median time to thrombosis in this study was 11 days, which was shorter than the 15 days post-surgery observed in White's study [7]. This discrepancy could be ascribed to our research's enhanced detection techniques by echocardiography during ICU stays, which likely increased our sensitivity for thrombus identification. These findings underscore the critical nature of the immediate post-Norwood period, particularly the first two weeks, as a high-risk term for thrombus.

Thrombus formation after the Norwood procedure has been reported to occur at various locations, reflecting the complex anatomical changes and postoperative physiological state inherent to the Norwood procedure [6,7]. Our study identified the SVC as the most common site for thrombosis, followed by the right atrium and the inferior vena cava. The reasons may include anatomical characteristics of relatively small vessels that are prone to decreased blood flow velocity and turbulence, decreased venous return in the upper body due to prolonged postoperative bed rest, drug administration via a central venous catheter that is generally inserted at the SVC, and stimulation of the vessel wall.

### 4.2 Etiology and risk factors for thrombus formation

The Norwood procedure results in dramatic changes in systemic and pulmonary blood flow patterns, which, coupled with the hyper-coagulable state, and the use of artificial devices such as a systemic-pulmonary shunt, create conditions conducive to thrombus formation [8]. In addition, the significant inflammatory response and potential endothelial dysfunction caused by this invasive procedure can promote thrombogenesis [9]. Heart failure, a common sequela of the Norwood procedure, results in poor perfusion and hemodynamic changes that further predispose patients to thrombosis. Thus, the multifaceted etiology, including surgical, physiologic, and patient-specific factors, increases the risk of thrombosis. Our analysis demonstrated that preoperative restrictive ASD was an independent risk factor for thrombus formation. Although atrioseptectomy was always performed at the Norwood procedure, preoperative restrictive ASD remained an increased risk for thrombus formation. The association between preoperative restrictive ASD and postoperative thrombosis likely reflects persistent alterations in atrial flow dynamics and endothelial function. Even after surgical septectomy, these patients may maintain pro-thrombotic changes in their atrial architecture and flow patterns. Additionally, the chronic pressure and volume overload associated with restrictive ASD may induce lasting changes in endothelial function and coagulation cascades that increase thrombotic risk in the postoperative period. These changes may be attributed to the flow stagnation of pulmonary venous return, turbulent blood flow in the right atrium, and peripheral circulatory failure associated with decreased systemic cardiac output [10].

While the long-term survival and complications between the MBTTS and the RVPAC have been extensively studied, the risk of early postoperative thrombosis has been less well reported [11,12]. Our findings showed that RVPAC patients may be at higher risk for early thrombotic events compared to MBTTS patients, especially for thrombus in the systemic veins. One explanation might be a different anticoagulation strategy between patients with a MBTTS and a RVPAC in our institute. We started intravenous administration of unfractionated heparin at a dose of 5000 IU/m2/day 6–8 h after admission to the ICU until removal of the central venous catheter. Thereafter, only patients with a shunt diameter of 4 mm or less received acetylsalicylic acid (3–5 mg/kg/day), and patients with a shunt diameter of more than 4 mm had no further anticoagulation. This might be an explanation for the increased risk of thrombus in RVPAC patients than in MBTTS patients. Another explanation may be that patients with a RVPAC are frequently associated with postoperative heart failure and low cardiac output syndrome because we prefer to use a RVPAC in patients with aortic atresia and small diameter of ascending aorta [4,13]. However, establishing a direct causal relationship between these parameters and thrombus formation requires a comprehensive evaluation of data, including other clinical parameters.

### 4.3 Future prospective

Thromboprophylaxis in the post-Norwood procedure remains a critical yet challenging aspect of patient management. The lack of high-level evidence has led to significant variability in prevention strategies across institutions and physicians. Drugs such as aspirin, warfarin, and low-molecular-weight heparin reduce the risk of thrombosis. However, targeted approaches are required because thrombus formation is still frequent [14]. The heterogeneity in reported incidence and occurrence of thrombus further complicates the development of universal protocols. This variability is often attributed to diverse patient populations and the evolution of surgical techniques over the past decades. Consequently, there is an urgent need for contemporary, well-designed prospective studies to provide a more nuanced understanding of thrombosis risk factors and timing specific to the post-Norwood period.

## 5 Limitations

The study has the distinct disadvantages of a retrospective nature and a single-center analysis with inherent bias. Our relatively small sample size limits the statistical power to detect clinically relevant differences between groups, potentially obscuring important associations or effects. While our analysis demonstrates an association between thrombus formation and adverse outcomes, causality cannot be established due to the retrospective design. Thrombus formation may be a marker rather than a cause of poor hemodynamic status, particularly in cases requiring ECMO support. Future prospective studies are needed to better understand this relationship. Missing data are likely to be associated with patient outcomes and may introduce bias in our findings.

## 6 Conclusions

In conclusion, this study reveals that post-Norwood thrombus formation is associated with adverse outcomes, which may reflect underlying hemodynamic compromise rather than a direct causal relationship, particularly in patients with restrictive ASD. These findings may emphasize the need for careful monitoring and potentially more aggressive thromboprophylaxis strategies, especially during the first 2 weeks after the Norwood procedure, with special attention to patients showing signs of hemodynamic deterioration.

## CRediT authorship contribution statement

**Alessandra Poppe:** Writing – original draft, Investigation, Data curation. **Muneaki Matsubara:** Writing – original draft, Methodology, Investigation, Formal analysis, Data curation. **Jonas Palm:** Writing – review & editing, Methodology, Investigation, Data curation. **Thibault Schaeffer:** Writing – review & editing, Visualization, Investigation, Formal analysis. **Takuya Osawa:** Writing – review & editing, Data curation. **Carolin Niedermaier:** Writing – review & editing, Data curation. **Paul Philipp Heinisch:** Writing – review & editing. **Nicole Piber:** Writing – review & editing. **Bettina Ruf:** Writing – review & editing, Validation, Project administration, Methodology, Investigation, Data curation. **Alfred Hager:** Writing – review & editing, Validation, Supervision, Investigation, Conceptualization. **Peter Ewert:** Writing – review & editing, Validation, Supervision, Methodology, Investigation, Conceptualization. **Jürgen Hörer:** Writing – review & editing, Validation, Supervision, Project administration, Conceptualization. **Masamichi Ono:** Writing – review & editing, Visualization, Supervision, Project administration, Investigation, Data curation.

## Funding statement

This study was supported by grants from the Förderverein des Deutschen Herzzentrums München.

## Declaration of competing interest

The authors declare that they have no known competing financial interests or personal relationships that could have appeared to influence the work reported in this paper.
