# Supplementary material for: Thrombus formation after the Norwood procedure: Incidence, risk factors, and its impact on late outcomes
Source: Int J Cardiol Congenit Heart Dis. 2025 Feb 15;20:100575. doi: 10.1016/j.ijcchd.2025.100575 (PMC12053980; doi:10.1016/j.ijcchd.2025.100575)
Supplement: Multimedia component 1 [file mmc1.docx]

**Supplementary Tables**

**Supplementary Table S1**

| Supplementary Table S1: Early postoperative data on coagulation | | | | |
| --- | --- | --- | --- | --- |
| **Variables: N(%) or median (IQR)** | **Total** | **Thrombus(+)** | **Thrombus(-)** | **p-value** |
| Number of patients | 360 | 42 | 318 |  |
| **APTT (s)** | | | | |
| Preoperative | 43 (37-50) | 42 (39-54) | 43 (37-49) | 0.336 |
| POD 1 | 53 (44-72) | 63 (46-77) | 52 (44-71) | 0.687 |
| POD 3 | 63 (49-81) | 69 (48-96) | 62 (49-78) | 0.150 |
| POD 7 | 65 (49-86) | 78 (54-102) | 63 (48-85) | **0.007** |
| POD 14 | 60 (44-85) | 69 (46-106) | 58 (43-82) | **0.027** |
| **PT-INR** | | | | |
| Preoperative | 1.2 (1.1-1.3) | 1.1 (1.1-1.3) | 1.2 (1.1-1.3) | 0.559 |
| POD 1 | 1.2 (1.1-1.3) | 1.2 (1.1-1.4) | 1.2 (1.1-1.3) | 0.741 |
| POD 3 | 1.2 (1.1-1.4) | 1.2 (1.1-1.4) | 1.2 (1.1-1.4) | 0.717 |
| POD 7 | 1.2 (1.1-1.3) | 1.2 (1.1-1.4) | 1.2 (1.1-1.3) | 0.677 |
| POD 14 | 1.2 (1.1-1.3) | 1.2 (1.1-1.3) | 1.2 (1.1-1.4) | 0.684 |
| **Antithrombin (%)** | | | | |
| Preoperative | 63 (54-72) | 62 (54-71) | 63 (54-72) | 0.468 |
| POD 1 | 67 (58-79) | 72 (54-82) | 67 (58-78) | 0.539 |
| POD 3 | 65 (54-76) | 66 (57-83) | 65 (54-76) | 0.818 |
| POD 7 | 65 (55-75) | 70 (56-80) | 64 (55-74) | 0.210 |
| POD 14 | 74 (63-85) | 73 (67-82) | 74 (62-85) | 0.609 |
| **Fibrinogen (mg/dL)** | | | | |
| Preoperative | 231 (200-286) | 213 (178-240) | 235 (201-288) | 0.095 |
| POD 1 | 224 (195-257) | 212 (158-236) | 225 (198-258) | 0.068 |
| POD 3 | 233 (178-300) | 198 (132-251) | 237 (183-301) | **0.010** |
| POD 7 | 247 (195-324) | 182 (146-255) | 261 (200-334) | **<0.001** |
| POD 14 | 247 (198-343) | 245 (189-337) | 247 (199-349) | 0.843 |

APTT: activated partial thromboplastin time; PT-INR: prothrombin time international normalized ratio
